# The Role of Nutritional Environment in *Cryptococcus gattii* Titan Cells’ Ultrastructure, Biophysical Properties, Molecular Features, and Virulence in Cryptococcosis

**DOI:** 10.3390/idr17040101

**Published:** 2025-08-16

**Authors:** Igor Avellar-Moura, Glauber R. de S. Araujo, Juliana Godoy, Vinicius Alves, Iara Bastos de Andrade, Juliana Soares, Bruno Pontes, Susana Frases

**Affiliations:** 1 Laboratório de Biofísica de Fungos, Instituto de Biofísica Carlos Chagas Filho, Universidade Federal do Rio de Janeiro, Rio de Janeiro 21941-902, Brazil; igoravellar@biof.ufrj.br (I.A.-M.); godoy0229@gmail.com (J.G.); viniciusalves@biof.ufrj.br (V.A.); iarabastos810@gmail.com (I.B.d.A.); 2 Laboratório de Pinças Ópticas, Instituto de Ciências Biomédicas, Universidade Federal do Rio de Janeiro, Rio de Janeiro 21941-902, Brazil; jussmp@biof.ufrj.br (J.S.); bpontes@icb.ufrj.br (B.P.); 3 Centro Nacional de Biologia Estrutural e Bioimagem, Universidade Federal do Rio de Janeiro, Rio de Janeiro 21941-902, Brazil; 4 Rede Micologia RJ, Fundação Carlos Chagas Filho de Amparo à Pesquisa do Estado do Rio de Janeiro, Rio de Janeiro 21941-902, Brazil

**Keywords:** *C. gattii*, titan cells, Neurobasal™ medium, virulence

## Abstract

Background/Objectives: *Cryptococcus gattii* presents a significant threat to healthy individuals. Titan cell formation, a key virulence factor, is influenced by the nutritional environment and plays a critical role in immune evasion and stress resistance. This study investigates the molecular and biophysical changes in titanized *C. gattii* cells grown in nutrient-rich Neurobasal™ medium, a potent inducer of titan cells. Methods: An integrative approach was used, combining scanning electron microscopy, optical tweezers, fluorescence microscopy, and physicochemical methods to analyze *C. gattii* cells grown in Neurobasal™ medium and minimal media. Results: Cells grown in Neurobasal™ medium exhibited significant differences compared to those grown in minimal media. These included a thicker and more defined polysaccharide capsule, enhanced capsule elasticity, and the secretion of more elastic polysaccharides. Furthermore, cells grown in the enriched medium showed reduced susceptibility to antifungals and delayed mortality in infection models. Conclusions: *C. gattii* adapts to nutritional cues by forming titan cells, thereby enhancing its pathogenicity. Targeting nutritional sensing pathways may offer novel therapeutic strategies against cryptococcal infections.

## 1. Introduction

*Cryptococcus gattii* is an emerging fungal pathogen that poses a significant threat to public health, causing severe infections in both immunocompetent and immunocompromised individuals. While *C. neoformans* is responsible for most cases, *C. gattii* accounts for 11–33% of cryptococcosis cases worldwide. This wide range reflects substantial geographic variation, with higher prevalence in certain endemic regions, as well as differences in study populations and diagnostic methods [1,2]. It is associated with cryptococcal meningitis and pulmonary cryptococcosis, presenting symptoms such as headaches, fever, neck stiffness, altered mental status, cough, and chest pain [3,4]. Initially restricted to tropical and subtropical regions, *C. gattii* has now expanded into temperate areas, including the Pacific Northwest of the United States and parts of Canada, raising concerns about its adaptability and potential for further spread. This geographic expansion underscores the urgent need for enhanced surveillance and public health interventions [5,6,7]. Treatment often requires prolonged antifungal therapy, typically with drugs like amphotericin B and flucytosine, both of which have significant side effects, complicating clinical management. The high mortality rate, especially with delayed treatment, and the risk of long-term neurological complications further emphasize the clinical importance of *C. gattii* [8].

A key factor in *Cryptococcus neoformans*/*gattii* complex virulence is the polysaccharide (PS) capsule, which inhibits phagocytosis and modulates host immune responses [9,10]. The capsule also contributes to biofilm formation, protecting fungal cells from environmental stressors and antifungal agents, thus complicating treatment [11]. Variations in capsule structure and size influence pathogenicity, with different morphotypes exhibiting distinct virulence profiles. Under capsule-inducing conditions, such as serum or starvation, the size and composition of the capsule can change, impacting the fungus’s ability to evade host defenses [11,12].

One notable morphological adaptation in *C. gattii* is the formation of titan cells—enlarged polyploid cells (up to 50–100 µm in diameter) with thickened capsules. Induced by specific environmental conditions, these cells enhance virulence through multiple mechanisms, such as resisting phagocytosis and modulating the immune response to a less protective Th2 profile [13,14,15,16]. Hypervirulent VGII strains from the Pacific Northwest outbreak exhibit an increased capacity for titan cell formation, potentially contributing to their elevated pathogenicity [13,14,15,16].

Despite advances in cryptococcal research, the precise effects of culture conditions on *C. gattii* capsule structure remain poorly understood. There is currently no consensus on the precise definition of titan cells, as various authors employ different size parameters and morphogenetic characteristics in their classifications [17]. In our study, we consider cells with a diameter greater than or equal to 15 μm as titan cells. We recently found that Neurobasal™ (NB) medium, commonly used for long-term neuronal culture, induces *Cryptococcus* titanization, enabling deeper investigation into how culture conditions influence capsule formation [17]. While the critical role of capsule in virulence is well established, further investigation is needed to uncover the modifications that occur under different environmental conditions and their impact on pathogenicity [12,18]. Understanding the triggers and mechanisms behind titan cell formation could provide novel therapeutic targets, potentially improving outcomes for patients with *C. gattii* infections, especially those caused by hypervirulent strains.

In this work, we investigated *C. gattii* titanization by examining capsular architecture and cell wall chitin under different media conditions, with a particular focus on the clinical relevance of the central nervous system (CNS) as a primary site of *Cryptococcus* infection. Neurobasal™ medium (NB), which provides an environment that mimics key aspects of the CNS encountered by the fungus during meningoencephalitis, was compared to minimal medium (MM). While NB offers a nutrient-rich condition that supports cellular activity, MM, a defined medium commonly used for *Cryptococcus* cultivation, imposes glucose and nutrient limitations, thereby inducing a starvation state and enabling the investigation of cellular responses to nutrient stress [17]. By establishing an in vitro model that reflects both conditions, we aimed to carefully examine how nutritional cues influence titan cell formation and capsule properties. Our integrative approach—combining microscopy, optical tweezers, and physicochemical analysis—revealed striking differences between cells grown in NB versus MM media. Titanized cells from NB exhibited distinct morphological and biophysical properties that likely contribute to immune evasion and antifungal resistance. These insights into *C. gattii*’s adaptive strategies suggest promising therapeutic targets that could transform management of these challenging infections.

## 2. Materials and Methods

### 2.1. Strain Information

The study employed the *Cryptococcus gattii* clinical isolate L25/01, kindly provided by Professor Daniel Assis Santos from the Department of Microbiology at the Institute of Biological Sciences (Federal University of Minas Gerais, Brazil).

### 2.2. Growth Conditions

To induce nutritional deprivation and promote polysaccharide (PS) capsule growth, Minimal Medium (MM) was used, consisting of 15 mM glucose, 10 mM MgO_4_S * 7 H_2_O, 29 mM KH_2_PO_4_, and 13 mM glycine in ultrapure Milli-Q^®^ water: Pharmaceutical Technology (MilliporeSigma Life Science Campus, Burlington, MA, USA), with a pH of 5.5 ± 0.1 (at 25 °C), as described by Zaragoza et al. [19]. Additionally, Neurobasal™ medium (NB) (Thermo Fisher Scientific, Waltham, MA, USA, Cat.# 21103049), supplemented with 2 mM L-glutamine (Gibco™, Cat.# 25030081), 1% penicillin/streptomycin (Gibco™, Cat.# 15140122), and B-27™ (Gibco™, Cat.# 17504044) supplement (Gibco, Thermo Fisher Scientific, Waltham, MA, USA), was investigated as an alternative medium for in vitro titan cell production, based on a previously established protocol by our group [17].

A pre-inoculum of *C. gattii* yeast was prepared in 10 mL of liquid Sabouraud medium (GranuCult^®^, Cat.# 1.08339, Merck KGaA, Darmstadt, Germany), incubated at 37 °C with continuous shaking at 150 rpm (approximately 5.59× *g*) for 24 h. Cells were collected by centrifugation at 6708× *g* for 10 min and quantified using a Neubauer chamber. In a 6-well plate, 2 mL of each test culture medium (MM and NB) was inoculated with 5 × 10^3^ fungal cells per mL and incubated at 37 °C with 5% CO_2(g)_ for 5 days.

### 2.3. Morphological Evaluation

To examine cell morphology in MM and NB, 10 µL of cultures were collected after 5 days of incubation, mixed with 10 µL of India ink, and observed under an Axiolab optical microscope (Carl Zeiss Microscopy, White Plains, NY, USA). Quantifications were performed on at least 150 cells using the ImageJ 1.53t bundled with Java 1.8.0_345 (64 bits) for macOS 15 (Sequoia) software (http://rsb.info.nih.gov/ij/) provided by the National Institutes of Health (NIH, Bethesda, MD, USA) [20,21].

Titan cells were defined as those with a cell diameter greater than or equal to 15 µm. This threshold was selected based on established criteria in the literature for *Cryptococcus* species [22,23], which have been primarily applied to *C. neoformans*. Recent studies, including that by Saidykhan et al. [13], have highlighted species-specific differences in titan cell characteristics in *C. gattii*, but the ≥15 µm diameter remains a widely accepted criterion. Only cells exceeding this size threshold were classified as titan cells in all analyses.

### 2.4. Scanning Electron Microscopy (SEM)

SEM was conducted following previously described procedures [24,25,26]. Briefly, cells of *C. gattii* were subjected to a washing procedure involving three rinses with phosphate-buffered saline (PBS) at a pH of 7.4 ± 0.1 (25 °C). Subsequently, the cells were fixed in a solution of 2.5% (v/v) EM-grade glutaraldehyde (Electron Microscopy Sciences, CAS #111-30-8) prepared in 0.1 M sodium cacodylate buffer (Electron Microscopy Sciences, CAS #124-65-2, Hatfield, PA, USA) for a duration of one hour at room temperature. After fixation, the cells were thoroughly rinsed three times with a 0.1 M sodium cacodylate buffer at pH 7.2 ± 0.1 (25 °C), which contained 0.2 M sucrose and 2 mM magnesium chloride (Merck Millipore, Darmstadt, Germany). To facilitate adherence, the cells were placed on 12 mm diameter round glass coverslips (Paul Marienfeld GmbH & Co. KG, Lauda-Königshofen, Germany) that had been pre-treated with a 0.01% solution of poly-L-lysine (Sigma-Aldrich Chemie GmbH, Taufkirchen, Germany, CAS #25988-63-0) for 20 min. Following adherence, the cells underwent a gradual dehydration process through a series of ethanol solutions (Merck Millipore, CAS# 64-17-5) series (v/v), specifically 30%, 50%, and 70% for 5 min each, followed by two rounds of 95% and 100% ethanol for 10 min each. The coverslips were subsequently dried using an EM DPC 300 critical point dryer (Leica Microsystems, Wetzlar, Germany) and mounted onto specimen stubs with a conductive carbon adhesive (Pelco Tabs™, Stansted, Essex, UK). The samples were sputter-coated with a 10 nm layer of platinum (Pt) using a Q150R Plus sputter coater (Quorum Technologies, Judges House, UK) and examined with a Quattro ESEM (Thermo Scientific™, Waltham, MA, USA) or Zeiss EVO 10.

### 2.5. Capsular Antigens and Chitin Quantification

Briefly, *C. gattii* cells (10^6^) yeast cells cultivated in MM and NB media were subjected to centrifugation at 6708× *g* for 5 min. The resulting pellet was then resuspended in a 4% (v/v) paraformaldehyde solution (Electron Microscopy Sciences, CAS #15700) prepared in phosphate-buffered saline (PBS), which consisted of 137 mM sodium chloride, 2.7 mM potassium chloride, 10 mM disodium hydrogen phosphate, and 1.8 mM potassium dihydrogen phosphate, adjusted to a pH of 7.4 ± 0.1 (25 °C). The cells were incubated at room temperature for 30 min to allow for fixation. Following the fixation process, the cells were washed twice with PBS and subsequently incubated with 1% bovine serum albumin (Sigma Aldrich, CAS #9048-46-8, St. Louis, MO, USA) in PBS for one hour at room temperature. The cells were then treated with the 18B7 monoclonal antibody at a concentration of 10 µg/mL for one hour at room temperature. The 18B7 mAb, a mouse IgG1, is known for its high affinity binding to glucuronoxylomannan (GXM) from various cryptococcal serotypes. After three washes with PBS, the cells were incubated with 10 µg/mL anti-mouse Alexa Fluor^®^ 546 secondary antibodies (Thermo Fisher Scientific, Cat # A-11030) for one hour at room temperature (25 °C). Following this incubation, the cells were washed with PBS and stained with 1% (w/v) UVITEX 2B in PBS (Polyscience Inc., CAS# 27344-41-8, Warrington, PA, USA) for one hour at room temperature. This was followed by four thorough washes with PBS to effectively remove the UVITEX 2B dye and minimize background staining. The cell suspensions were then mounted on glass coverslips and examined using a ZEISS Elyra PS.1 microscope (Carl Zeiss Microscopy, White Plains, NY, USA). Images were captured using the corresponding software packages [ZEN blue 3.6 (Carl Zeiss Microscopy GmbH), White Plains, NY, USA] and the images were subsequently subjected to analysis using the ImageJ 1.53t bundled with Java 1.8.0_345 (64 bits) for macOS 15 (Sequoia) software (http://rsb.info.nih.gov/ij/) provided by the National Institutes of Health (NIH, Bethesda, MD, USA). For fluorescence quantification, cells were adjusted to a concentration of 1 × 10^4^ cells/mL and analyzed using a flow cytometer (BD LSRFortessa™ X-20, BD Biosciences, San Jose, CA, USA) with excitation at 350 nm and emission at 435 nm for UVITEX 2B and excitation peak at 561 nm and an emission peak at 572 nm for Alexa Fluor^®^ 546. The untreated population was mapped without labeling, and this gate was used for both treated and untreated cells. The population (*n* = 10,000 events) was analyzed for size and fluorescence intensity, with results expressed in Mean Fluorescence Intensity (MFI) = [(∑_i_ I_i_)/n], where ∑_i_ represents the sum over all cells/particles; I_i_ is the fluorescence intensity of the cell/particle; n is the total number of cells/particles measured. Additionally, cells were observed under an Axio Observer fluorescence microscope (Carl Zeiss Microscopy, Thornwood, NY, USA) for morphological assessment.

### 2.6. Young’s Modulus Measurements with Optical Tweezers

To measure the Young’s modulus of the *C. gattii* capsules, glass-bottomed dishes were coated with 10 μg/mL of mAb 18B7, as previously described [27]. A 200 μL suspension containing 10^4^ *C. gattii* cells in PBS was added to the dishes and incubated for 1 h at room temperature. After washing with PBS to remove any nonadherent cells, polystyrene microspheres with a diameter of 3 µm (Polyscience Inc., CAS# 9003-53-6, Warrington, PA, USA) were introduced to the dish, and the samples were placed in the Optical Tweezers (OT) system. To assess the Young’s modulus (expressed in Pascals (Pa) or Newtons per square meter (N/m^2^)) of the PS capsule of *C. gattii*, a polystyrene bead was captured with the OT and pressed against the capsule for 1 min to attach the bead to the fungal capsule. The microscope stage was then moved at a controlled speed (1.000 ± 0.002 μm/s), causing the attached bead to shift its equilibrium position in the trap and deform the fungal capsule. The entire process was recorded using an Infrared Charged–Coupled Device (CCD) Hamamatsu Photonics C2400 video camera (Hamamatsu, Japan), and ImageJ software [Fiji (ImageJ 2.14.0/1.54f, NIH, Bethesda, MD, USA)] was used to determine the change in equilibrium position of the trapped bead over time. These data were then used to calculate Young’s modulus based on previously established model [27].

### 2.7. Extraction and Concentration of Secreted Polysaccharides

For secreted PS extraction, cells were cultured in Sabouraud dextrose medium, centrifuged, and resuspended in MM and NB media. After incubation, supernatants were concentrated using ultrafiltration, and PS was quantified using the phenol–sulfuric method [28,29].

### 2.8. Dynamic Light Scattering (DLS), Zeta Potential (ζ), and Conductance Measurements

Cells and secreted PSs were prepared following the method outlined by Frases S. et al. [30]. Zeta potential (ζ) measurements were performed using a NanoBrook Omni with NanoBrook Particle Solutions software version 3.6.0.7136 (Brookhaven Instruments Corp., Holtsville, NY, USA), with ten measurements taken for each sample at 25 °C. Additionally, 10 mg/mL secreted PS solutions from each of the experimental conditions were used to determine the effective diameter of the PS fibers [30].

### 2.9. Passive Micro-Rheology

The viscoelastic properties of 10 mg/mL secreted PS solutions in deionized water were analyzed. Values for viscous modulus (G″), elastic modulus (G′), and complex viscosity (η*) were obtained using polystyrene microspheres with a diameter of 1 µm (Polyscience Inc., Warrington, PA, USA, CAS# 07310-15) as probes. Deionized water was used as the experimental control. Measurements were performed on the NanoBrook Omni for both experimental conditions, which were MM- and NB-derived [31,32].

### 2.10. Antifungal Activity Assessment

Minimal Inhibitory Concentrations (MICs) were determined using the broth microdilution technique, adhering to the guidelines set by the Brazilian Committee on Antimicrobial Susceptibility Testing (BrCAST) with modifications. Fluconazole (Sigma-Aldrich Co., St. Louis, MI, USA, CAS# 86386-73-4) and amphotericin B (Sigma-Aldrich Co., USA, CAS# 1397-89-3) were serially diluted (ranging from 20 to 0.03 µM) in MM and NB media in 96-well plates. The fungal inoculum was prepared as previously described. Plates were incubated at 37 °C with 5% CO_2(g)_ for 48 h. MICs were identified as the lowest concentration of fluconazole and amphotericin B that completely inhibited fungal growth, indicated by the absence of visible turbidity. Controls included cryptococcal cells cultured without drugs and culture medium without fungal cells.

### 2.11. Mice Survival

In this study, female BALB/c mice (*Mus musculus*), aged 6–8 weeks and weighing 20–25 g, acclimated for 7 days before experimentation. Animals were housed in plastic cages with white wood chips for bedding in a specific-pathogen-free facility, under controlled conditions (12 h light/dark cycle, 22 ± 1 °C, 40–60% humidity). Mice had ad libitum access to standard rodent chow and filtered water. Mice were infected with *C. gattii* strains cultured in both MM and NB for 5 days at 37 °C with 5% CO_2(g)_. Infection was established via intratracheal instillation of 50 µL suspension containing 10^6^ CFU/mL of *C. gattii*. Animals were monitored daily for survival, weight loss, respiratory distress, and behavioral changes. All procedures were approved by the Ethics Committee on the Use of Animals (CEUA) of the Centro de Ciências da Saúde (CCS) of the Universidade Federal do Rio de Janeiro (UFRJ) (protocol #022/24), registered with Conselho Nacional de Controle de Experimentação Animal—CONCEA (process #01200.001568/2013-87).

### 2.12. Data Analyses

Statistical analyses were performed using GraphPad Prism 10.4.1 (627) software for Windows 11 (Version 24H2). Appropriate parametric or non-parametric tests were applied after verifying data normality with the Shapiro–Wilk test, where a *p*-value of less than 0.05 was considered statistically significant [33,34]. The statistical analyses performed for each experiment are specified in the respective figure legends.

## 3. Results

### 3.1. Nutrition Influences C. gattii Morphology, Capsule Formation, and Titan Cell Development

**Figure 1:**
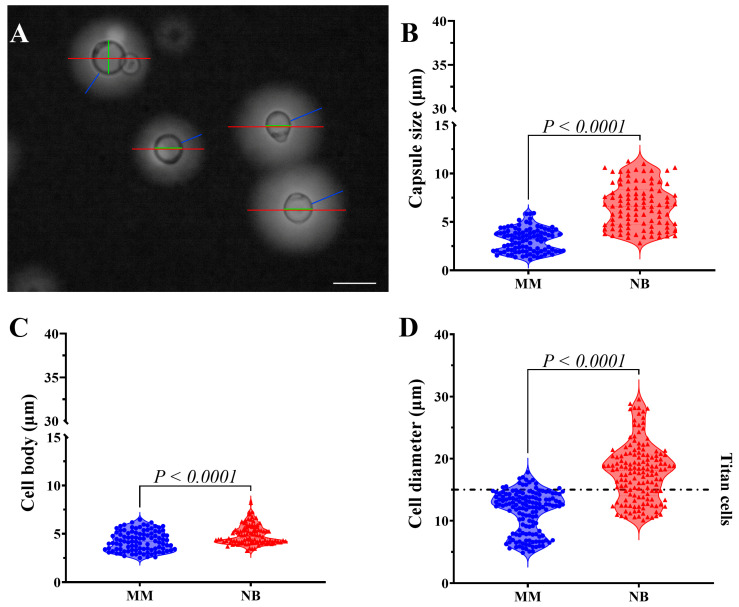
Morphometric analysis of *C. gattii* reveals nutrient-dependent changes in cell size and capsule development. (**A**) Brightfield optical microscopy of *C. gattii* stained with India ink. India ink is used to illustrate the morphometric measurements of *C. gattii*. The red line represents the total diameter of the cell (cell body plus PS capsule), while the green line indicates the cell body (a sphere with a refractive boundary). The blue line measures the PS capsule, which consists of the cell wall adjacent to the end of the birefringent halo. Scale bar 10 µm. (**B**) Measurements of the capsular size of 100 random cells revealed a significant increase in yeasts grown in Neurobasal™ medium (NB). In Minimal Medium (MM), the average capsular size was 3.116 ± 1.187, while in NB, it was 6.644 ± 2.232. (**C**) Measurements of the cell body indicated a significant increase in yeasts grown in NB medium. In MM, the average cell body size was 4.253 ± 1.015, while in NB, it was 4.871 ± 0.9705. (**D**) The cell diameter measurements indicate that 74% of the yeasts (with average sizes of 11.40 ± 3.366 µm and 17.80 ± 4.626 µm) have dimensions equal to or greater than 15 µm. The dotted line parallel to the *X*-axis indicates the limit of titan cells. Statistical significance was determined using a two-tailed *t*-test.

To investigate nutritional effects on *C. gattii* morphology, we compared cells grown in NB and MM. Our group previously identified NB as a potent inducer of *Cryptococcus* titanization [17]. Morphometric analysis confirmed *C. gattii* cells in NB had significantly larger capsules and total cell diameters than those in MM (Figure 1A–D). Notably, 74% of NB cells exceeded the 15 μm titan cell threshold, compared to only 9% of MM-grown cells (Figure 1D).

**Figure 2:**
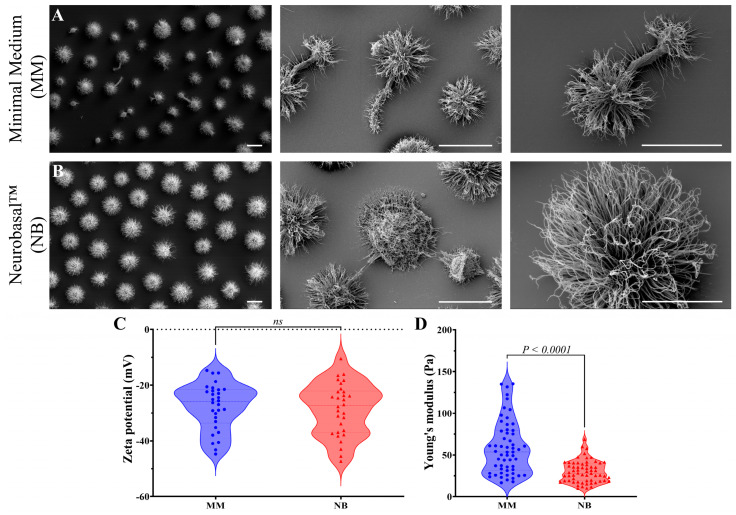
Nutritional environment influences on *C. gattii* morphology and capsule properties. Scanning electron microscopy reveals distinct morphological differences between cells grown in MM and NB. (**A**) MM-cultured cells showing irregular morphology, poor capsule development, and pseudo-hyphal arrangements. (**B**) NB-cultured cells displaying uniform spherical shape, larger size, and well-defined polysaccharide capsules with symmetrical fibril distribution. (**C**) Zeta potential analysis showing similar mean surface charge values but greater variability in NB-grown cells. (**D**) Biomechanical analysis using optical tweezers demonstrating significantly higher capsule elasticity (Young’s modulus) in MM-grown cells compared to NB-grown cells, indicating nutrient-dependent differences in capsule mechanical properties. The scale bars represent 10 μm. Statistical significance was determined using a two-tailed *t*-test.

Ultrastructural analysis with scanning electron microscopy (SEM) showed distinct morphological features between cells cultured in MM and NB (Figure 2). Cells in MM presented irregular shapes, poor capsule development, and abnormal budding patterns, including pseudo-hyphal arrangements (Figure 2A). In contrast, NB-grown cells were uniformly spherical, significantly larger, and exhibited well-defined, robust PS capsules with dense fibrils radiating symmetrically from the cell body (Figure 2B).

Next, Zeta potential analyses evaluated the surface charge properties of *C. gattii* cells under different growth conditions, providing insights into how the nutritional environment influences cell surface characteristics and their association with titan cell formation. Zeta potential measurements revealed similar mean surface charge between MM (−44.71 to −14.68 mV) and NB conditions (−47.34 to −10.45 mV) (Figure 2C). However, NB-cultured cells displayed greater variability in surface charge distribution. This predominantly negative charge across the conditions reflects capsular anionic polysaccharides, while the broader distribution in NB-grown cells suggests increased heterogeneity in capsule composition, potentially related to their enlarged capsule size.

Biomechanical analysis using optical tweezers showed significant differences in capsule elasticity (Figure 2D). MM-grown cells had a higher Young’s modulus (58.88 ± 32.05 Pa) than NB-grown cells (29.98 ± 12.90 Pa), with a broader range of values in MM cells (from 16.72 to 135.3 Pa) compared to the more constrained range in NB cells (from 9.37 to 68.70 Pa). Capsule stiffness correlates with morphological differences, indicating that nutrient availability influences both capsule size and mechanical properties.

**Figure 3:**
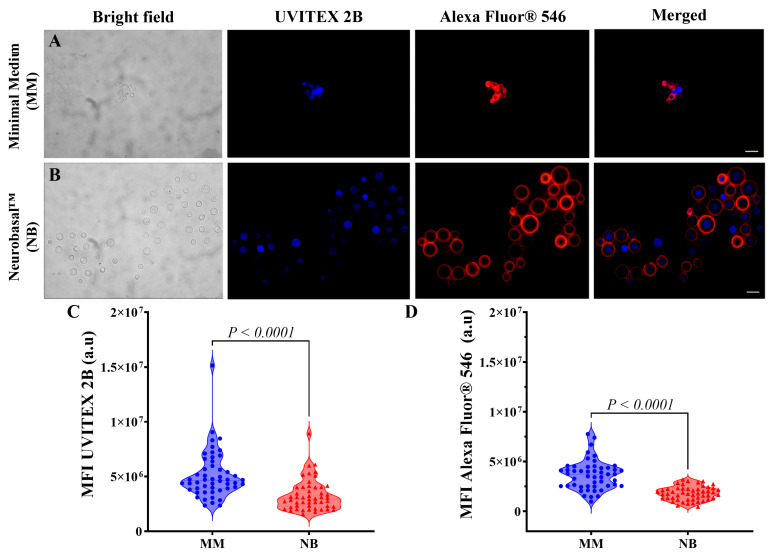
Nutritional conditions influence *C. gattii* cell wall composition and capsule architecture. Immunofluorescence microscopy with chitin-specific staining (blue—UVITEX 2B) and anti-capsular antibody 18B7 (red—Alexa Fluor^®^ 546) reveals distinct phenotypes: (**A**) MM-grown cells showing compact morphology with chitin localized to cell wall and minimal capsular extension; (**B**) NB-grown cells displaying enlarged dimensions with pronounced capsular expansion beyond well-defined chitin rings. Flow cytometry analysis demonstrates the following: (**C**) significantly higher chitin content in MM-grown cells compared to NB-grown cells; (**D**) higher capsular material concentration in MM-cultured cells despite their smaller overall capsule size, indicating nutritionally regulated differences in capsule density and architecture that correlate with mechanical properties. Statistical significance was determined using a two-tailed *t*-test.

Immunofluorescence using chitin staining (UVITEX 2B) and anti-capsular antibody 18B7 showed distinct phenotypes between the media (Figure 3). MM-grown cells exhibited compact morphology with localized chitin and minimal capsular extension (Figure 3A), while NB-grown cells displayed enlarged dimensions with pronounced capsular extension, forming prominent halos around well-defined chitin rings (Figure 3B). Merged images confirmed this differential organization (Figure 3A,B), correlating with the optical tweezers analysis, showing that MM cells have compact, rigid capsules while NB cells develop flexible, expanded capsules.

Flow cytometry measurements of Mean Fluorescence Intensity (MFI) demonstrated that cells grown in MM exhibited substantially higher chitin content, as detected by UVITEX 2B staining, compared to cells cultured in NB. The MFI values for MM-grown cells ranged from approximately 2.30 × 10^6^ to 1.50 × 10^7^ arbitrary units (a.u.), with a mean of 5.10 ± 2.20 × 10^6^ a.u. In contrast, NB-grown cells displayed significantly lower chitin-associated fluorescence, with MFI values predominantly between 1.5 × 10^6^ and 8.9 × 10^6^ a.u. (mean 3.30 ± 1.40 × 10^6^ a.u.) (Figure 3C). Similarly, capsular material quantification using anti-capsular antibodies conjugated with Alexa Fluor^®^ 546 demonstrated significantly higher fluorescence intensity in MM-cultured cells compared to NB-cultured cells (Figure 3D). The MFI values for capsular material in MM-grown cells ranged from approximately 0.98 × 10^6^ to 7.80 × 10^6^ a.u., with a mean of approximately 3.60 ± 1.50 × 10^6^ a.u., while NB-grown cells exhibited lower values predominantly between 0.46 × 10^6^ and 3.20 × 10^6^ a.u., with a mean of approximately 1.80 ± 0.68 × 10^6^ a.u.

### 3.2. Rheological and Structural Alterations in C. gattii Secreted Polysaccharides

**Figure 4:**
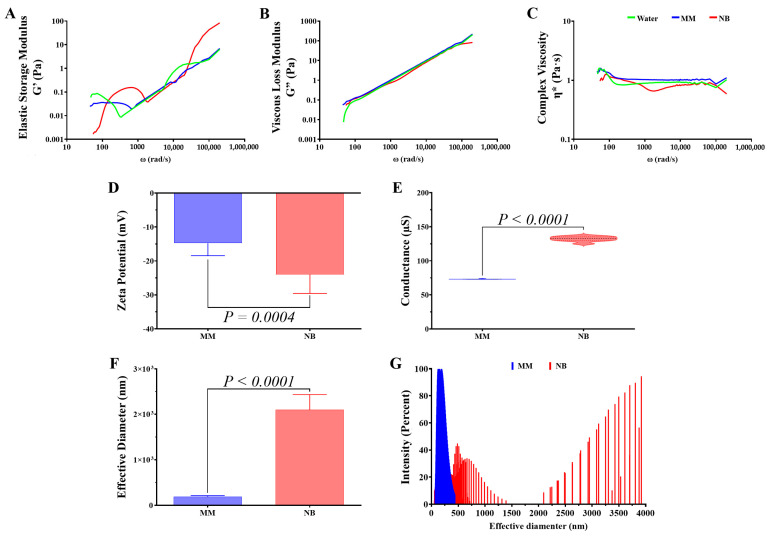
Rheological and physicochemical properties of *C. gattii* secreted polysaccharides vary with nutritional environment. (**A**) Elastic storage modulus (G′) showing frequency-dependent behavior with higher values in NB-secreted PS at intermediate frequencies. (**B**) Viscous loss modulus (G″) profiles displaying similar patterns between media. (**C**) Complex viscosity (η*) measurements revealing slight reductions in NB at intermediate frequencies. (**D**) Zeta potential analysis demonstrating significantly (*p*-value = 0.0004) more negative surface charge in NB-secreted PS compared to MM-secreted PS. (**E**) Electrical conductance measurements showing higher conductivity in NB-secreted PS. (**F**) Particle size analysis revealing dramatically larger PS particles in NB medium (12-fold increase). (**G**) Size distribution profiles indicating homogeneous small particles in MM versus bimodal distribution in NB-secreted PS. Statistical significance was determined using a two-tailed *t*-test.

Following the observation of substantial alterations in the capsular PS structure of *C. gattii*, we sought to determine whether these changes were also reflected in the PS secreted by this pathogenic fungus when cultured in MM compared to NB (Figure 4). Rheological analysis revealed the frequency-dependent behavior of elastic storage modulus (G′) (Figure 4A) and viscous loss modulus (G″) (Figure 4B) in both media, with secreted PS from NB showing higher G′ values at intermediate frequencies (100–10,000 rad/s), suggesting a more rigid structure with greater energy storage capacity. Complex viscosity (η*) and viscous loss modulus (G″) profiles were similar between media, with slight reductions in NB at intermediate frequencies (Figure 4B–C). The Zeta potential of secreted PS from MM (−14.79 ± 3.66 mV) was significantly less negative than those from NB (−23.99 ± 5.58 mV), indicating alterations in surface charge properties (Figure 4D). Electrical conductance measurements further differentiated the samples, with mean values of 73.10 ± 0.32 μS for MM-secreted and 132.20 ± 3.62 μS for NB-secreted PS (Figure 4E). Most strikingly, particle size analysis revealed that secreted PS from MM had a mean diameter of 192.30 ± 22.09 nm, while those from NB were approximately 12-fold larger (2098.00 ± 335.70 nm) (Figure 4F). Size distribution analysis showed a homogeneous population of small particles for MM-secreted PS versus a bimodal distribution for NB-secreted PS, with populations around 500 nm and above 3000 nm (Figure 4G). These results demonstrate that the nutritional environment significantly influences the physicochemical, rheological, and structural properties of PS secreted by *C. gattii*, which may have important implications for pathogen virulence in different host niches.

### 3.3. Culture Conditions Affect C. gattii Titan Cell Formation and Antifungal Susceptibility

The evaluation of antifungal agents (fluconazole and amphotericin B) against *C. gattii* grown in different culture media demonstrated distinct susceptibility patterns. Antifungal susceptibility tests showed significant medium-dependent variations in *C. gattii*’s response to antifungal agents. While fluconazole resistance remained consistent (MIC > 32 μg/mL) regardless of culture medium, amphotericin B susceptibility varied significantly. *C. gattii* cultured in MM showed substantially higher susceptibility to amphotericin B (MIC between 0.125–0.250 μg/mL), when compared to NB (MIC = 1 μg/mL), representing a 4- to 8-fold difference. These findings collectively demonstrate that culture conditions influence not only titan cell formation but also antifungal susceptibility profiles, particularly for amphotericin B.

### 3.4. Culture Environments Influence C. gattii Titan Cell Formation and Virulence

**Figure 5:**
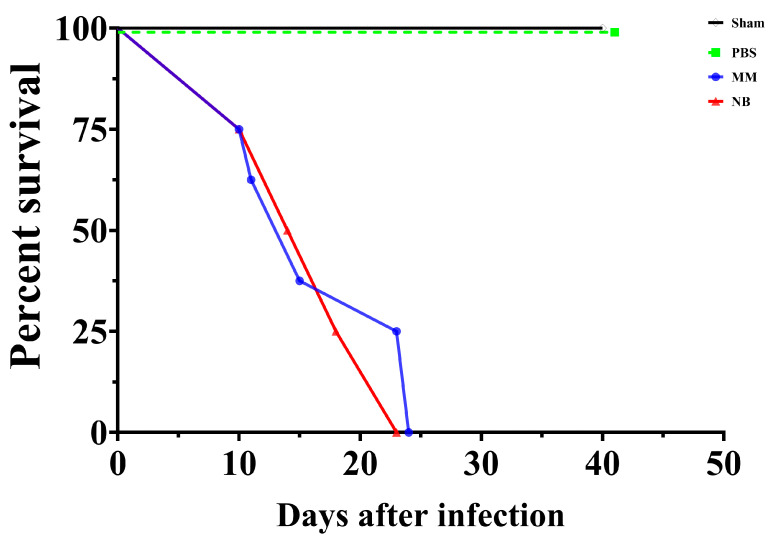
Nutritional environment influences *C. gattii* virulence in a murine survival model. Kaplan–Meier survival curves of mice infected with *C. gattii* cultured under different conditions. Control groups (Sham and PBS, black and green lines) maintained 100% survival throughout the 50-day observation period. Mice infected with MM-grown *C. gattii* (blue line) showed more rapid mortality, reaching 50% at approximately day 15, while the NB-grown *C. gattii* group (red line) reached the same threshold around day 18. Both infected groups reached 0% survival by day 23, with the delayed mortality curve in the NB group. Statistical analysis was performed using the log-rank (Mantel–Cox) test, and no statistically significant difference was observed between the infected groups. Each group consisted of *n* = 8 mice.

The survival analysis of mice infected with *C. gattii* cultured in different nutritional environments revealed differences between conditions (Figure 5). Control groups (Sham and PBS) maintained 100% survival throughout the 50-day observation period, confirming the experimental validity (black and green lines). Mice infected with *C. gattii* cultured in MM showed a more rapid decline in survival compared to those infected with NB-cultured *C. gattii* (blue line). Although both infected groups eventually reached 0% survival by approximately day 23, and the log-rank (Mantel–Cox) test did not reveal a statistically significant difference between the survival curves, a modest difference in the timing of mortality was observed. The MM group reached 50% mortality at approximately day 15, while the NB group reached the same threshold around day 18 (red line). This 3-day delay in the NB group suggests that NB-induced titanization may have influenced the virulence profile of *C. gattii*. These findings highlight how nutrient environment may influence titan cell development in *C. gattii*, which in turn affects host–pathogen interactions and disease progression in the murine model.

## 4. Discussion

Titan cells represent a critical morphological adaptation in *Cryptococcus* species that enhances virulence and promotes pathogenesis [19,35]. These enlarged cells, characterized by size (≥15 μm), polyploidy, and thickened capsules, provide advantages during infection by resisting phagocytosis, altering immune responses, and potentially serving as reservoirs for genetic diversity [36,37]. In *C. gattii*, titan cell formation is particularly significant for establishing infections in otherwise healthy hosts [38,39]. While extensively studied in *C. neoformans*, the regulation and functional significance of titan cells in *C. gattii* remain less characterized, especially regarding environmental triggers [13].

Our study provides evidence that nutritional environment significantly influences *C. gattii* morphology, capsule formation, surface properties, and titan cell development. Nutrient-rich NB medium induced higher titan cell formation compared to MM, suggesting specific nutritional components trigger this transition [17]. This finding aligns with previous observations in *C. neoformans*, indicating that *C. gattii* may possess distinct regulatory mechanisms for titan cell formation in response to environmental cues [13,17,40,41]. Scanning electron microscopy revealed that MM-cultured cells exhibited irregular morphology with poorly developed capsules, while NB-cultured cells presented uniform spherical shapes with thicker, well-defined polysaccharide capsules, characteristic of titan cells. This transition likely influences pathogen interactions with host tissues and immune cells, as enlarged titan cells with enhanced capsular structures resist phagocytosis, alter cytokine responses, and promote dissemination across the blood–brain barrier [13,14,40].

Morphometric analysis confirmed significant differences in capsule size, body size, and total cell size between growth conditions. NB-grown cells exceeded the ≥15 μm threshold for titan cell classification, associated with enhanced virulence and immune evasion [19]. The substantial increase in capsule dimensions (approximately 2.5-fold) in nutrient-rich conditions underscores the importance of environmental cues in modulating this key pathogenicity determinant [17]. Dambuza et al. [36] established that titan cell formation responds to environmental signals, including nutrient availability, while Hommel et al. [40] demonstrated nitrogen and carbon availability are critical for titan cell induction.

The capsule is a critical virulence factor protecting *Cryptococcus* from phagocytosis and oxidative stress. The results suggest that nutrient availability directly influences titan cell formation, immune evasion, and infection establishment. Titan cells exhibit significantly enlarged capsules that contribute to phagocytosis evasion [35] and enhanced oxidative stress resistance [42]. These cells resist phagocytosis by macrophages and neutrophils [37] and modulate host immune responses [43]. Recent research identified specific molecular pathways involved in titanization, including cAMP-PKA signaling [44] and transcription factor Rim101 [23].

Zeta potential analysis revealed comparable mean surface charge values between conditions, but greater variability in NB-grown cells. This broader distribution suggests nutrient-rich conditions influence cell surface charge heterogeneity in titan cells, potentially reflecting variations in capsule composition. The predominantly negative surface charge observed is consistent with anionic PSs in the cryptococcal capsule [45]. Greater heterogeneity in NB conditions may reflect variations in surface charge density associated with expanded capsular architecture, potentially enhancing immune evasion capabilities [45,46].

Optical tweezers analysis revealed that MM-grown cells exhibited higher Young’s modulus values compared to NB-grown cells, indicating greater capsular rigidity in nutrient-limited conditions. This mechanical disparity correlates with differences in capsular architecture, with MM-cultured cells displaying compact capsules and NB-cultured cells exhibiting expanded structures. The inverse relationship between capsular extension and mechanical rigidity suggests titan cell architecture influences biophysical properties of *C. gattii* [47]. The reduced rigidity in titan cell capsules may enhance survival, as more elastic capsules could better absorb mechanical stresses [48]. Importantly, previous studies from our group have demonstrated that increased capsule elasticity can hinder the formation of a stable phagocytic synapse, thereby reducing the efficiency of macrophage engulfment and contributing to immune evasion [26,27,47,48]. A more elastic capsule may also facilitate deformation of titan cells during tissue penetration and dissemination and can affect the diffusion of host antimicrobial molecules and antibodies. These findings highlight that, beyond capsule size, the biophysical properties of the cryptococcal capsule are critical determinants of pathogenesis.

Differences in capsule elasticity have been associated with variations in PS organization and packing density [49], potentially influencing complement deposition and phagocytosis efficiency [50]. More elastic capsules might allow greater deformation during interactions with phagocytes [35,51] and affect the diffusion of host antimicrobial molecules [52].

Immunofluorescence microscopy revealed MM-grown cells exhibited compact morphology with concentrated chitin localization and minimal capsular extension, while NB-grown cells displayed significantly enlarged dimensions and pronounced capsular expansion. Quantitative analysis showed MM-grown cells had higher chitin content and capsular stained material, despite compact morphology. These findings suggest differential regulation of cell wall and capsular components during titan cell formation. Increased chitin content is associated with cell wall integrity and stress resistance [53], while reduced chitin in NB-grown titan cells suggests the reorganization of cell wall components, potentially altering immunogenicity. The higher fluorescence intensity of capsular material in MM-grown cells suggests more densely packed capsule structure, while expanded but less intense stained capsule in NB-grown titan cells indicates more diffuse arrangement of capsular PS [54]. These structural differences may influence the accessibility of capsular epitopes to host immune receptors [46,55]. It is also important to consider that the observed lower chitin signal in NB-grown cells, as detected by UVITEX 2B staining, may not solely reflect a true reduction in chitin content. The expanded capsule and altered cell wall architecture characteristic of NB-grown titan cells could influence the accessibility of the chitin-binding dye. Changes in cell wall permeability or the masking effect of the enlarged capsule may partially restrict UVITEX 2B penetration, leading to a reduced fluorescence signal despite the actual chitin content. Therefore, the lower chitin signal in NB-grown cells may result from both potential differences in chitin synthesis or organization and altered accessibility due to the unique structural properties of titan cells.

Despite the larger capsule size observed in NB-grown cells, the Mean Fluorescence Intensity (MFI) for capsular antigen detection was lower compared to MM-grown cells. This apparent discrepancy likely reflects changes in capsule architecture and/or composition. Larger capsules can exhibit increased density or altered organization of polysaccharide fibers, which may result in epitope masking and reduced accessibility of antibody-binding sites, as previously reported for *Cryptococcus* species [50,54,55]. Environmental conditions are known to modulate the structure and antigenic properties of the *cryptococcal* capsule, affecting the exposure and recognition of specific epitopes by antibodies [50,54]. Additionally, variations in the relative abundance of capsule components, such as glucuronoxylomannan (GXM) and galactoxylomannan (GalXM), may occur under different growth conditions, further influencing immunoreactivity [12,56]. Although we did not directly quantify the (GXM/GalXM) ratio in this study, our findings are consistent with those of previous reports, showing that capsule expansion can be accompanied by changes in density and epitope accessibility, rather than a simple increase in antigen content [12,50,54,55]. Further biochemical analyses will be important in future studies to directly assess changes in capsule composition under different nutritional conditions.

Analysis of secreted PS revealed medium-dependent modifications in rheological properties. NB-cultured cells produced PS with higher elastic storage modulus values, suggesting a more rigid structure. Particle size distribution differed dramatically between conditions [30,57], with NB-derived PS showing bimodal distribution indicating complex aggregates. These larger aggregates can inhibit phagocytosis and complement activation [56], potentially enhancing immunomodulatory properties [58]. Such modifications may influence biofilm formation [59] and alter antigen presentation to host immune receptors [60].

NB-grown titan cells showed 4–8-fold reduced susceptibility to amphotericin B, highlighting the clinical relevance of nutritionally induced titan cell formation. This reduced sensitivity is consistent with findings by Gerstein et al. [37], who reported titan cells exhibit increased resistance to multiple antifungal agents. Amphotericin B targets ergosterol in fungal cell membranes, and reduced susceptibility may reflect changes in membrane composition or accessibility [61]. The expanded capsule likely creates a barrier limiting antifungal penetration [19]. This clinically relevant shift in susceptibility could impact treatment outcomes, particularly in the anatomical sites where titan cells predominate [35]. Titan cells can produce normal-sized daughter cells that retain parental resistance phenotypes, establishing a reservoir of less susceptible fungal cells [37]. Future studies will be important for investigating whether these nutritional conditions also impact membrane composition and ergosterol content, which could further contribute to the observed changes in antifungal susceptibility.

Survival analysis in our murine model revealed a modest delay in mortality for mice infected with NB-grown *C. gattii* compared to those infected with MM-grown cells. Although both groups ultimately reached 0% survival, the observed difference in median survival time (approximately 3 days) suggests that the culture conditions promoting titan cell formation may influence the early progression of infection. However, this effect was limited and did not translate into improved overall survival. These findings indicate that, while titan cell formation and associated phenotypic changes—such as increased cell size and altered surface properties—may transiently modulate host–pathogen interactions, they do not appear to significantly alter the outcome of infection in this model. This is consistent with previous reports that titan cells can delay dissemination and initial immune responses due to their size [35,62], but ultimately give rise to normal-sized daughter cells that are capable of sustaining infection [37]. Thus, nutritional priming and titanization may impact the timing and dynamics of early infection events without preventing eventual lethal disease [22].

We acknowledge several limitations in our study that should be considered when interpreting the results. Although our detailed analyses focused primarily on a single clinical isolate (L25/01), preliminary experiments including additional *C. gattii* strains, demonstrated that the titanization effect of Neurobasal™ medium was consistent. Our experiments were conducted using in vitro models, which are essential for dissecting specific mechanisms and environmental influences but do not fully recapitulate the complexity of the host environment. Factors such as immune interactions, tissue architecture, and dynamic nutrient availability in vivo may influence titan cell formation and antifungal susceptibility in ways that are not captured by our current system. Moreover, while the Neurobasal™ medium proved to be effective in inducing titanization, it may not precisely mimic the nutritional conditions encountered by *Cryptococcus* during infection [13,23]. The precise molecular mechanisms and signaling pathways underlying the observed effects remain to be fully elucidated. While our results highlight correlations between medium composition and titan cell formation, further studies employing genetic, pharmacological, and in vivo approaches will be necessary to validate and expand upon these observations [63].

Despite these limitations, our findings provide important insights into the environmental regulation of titan cell formation and underscore the urgent need for new methodologies that more accurately model the host environment and the signals that drive titanization. Developing such approaches will be crucial for advancing our understanding of cryptococcal pathogenesis and for identifying novel therapeutic strategies targeting this unique morphotype.

## 5. Conclusions

Collectively, our findings demonstrate that the nutritional environment is a critical determinant of *C. gattii* titan cell formation, influencing key phenotypic traits such as cell size, capsule production, and surface properties. While these adaptations may have implications for pathogenesis, our survival studies indicate that titanization alone does not significantly alter the ultimate outcome of infection in the murine model used, as both groups presented the same mortality rate. The modest delay in mortality observed suggests that titan cell formation may transiently affect the early course of infection, potentially by modulating initial host–pathogen interactions. However, further studies are needed to clarify the precise role of titan cells in virulence and disease progression in vivo. From a therapeutic perspective, targeting the signaling pathways involved in nutritional sensing and titan cell formation remains a promising avenue, but its impact on clinical outcomes requires additional investigation. Our study highlights the importance of the nutritional environment in shaping *C. gattii* morphology and provides a foundation for future research into the mechanisms and consequences of titan cell formation during infection.

## Data Availability

The data supporting the findings of this study are available from the corresponding author upon reasonable request.
